# Erosion of a right ventricular pacer lead into the left chest wall

**DOI:** 10.1186/s40792-020-00999-3

**Published:** 2020-10-06

**Authors:** Michael J. Herr, J. Macy Cottrell, H. Edward Garrett Jr., Darryl S. Weiman

**Affiliations:** 1 College of Medicine, University of Tennessee Health Science Center, 910 Madison Ave. 10th floor, Memphis, TN 38103 USA; 2 Department of Anatomy and Neurobiology, University of Tennessee Health Science Center, 855 Monroe,5th floor, Memphis, TN 38103 USA; 3 Department of Surgery, University of Tennessee Health Science Center, 910 Madison Ave. 2nd floor, Memphis, TN 38103 USA

**Keywords:** Chest wall, Rib perforation, Pacing lead

## Abstract

**Background:**

Erosion of a pacer lead into the chest wall may result in pericardial effusion with cardiac tamponade. Free rupture into the pleura or mediastinum can result in hypotension and cardiac arrest.

**Case presentation:**

We report a unique case of a right ventricular pacer lead which eroded through the right ventricle into the left chest wall and penetrated a rib. The patient presented with a tender chest wall mass without pericardial or pleural effusion. The segment of rib which the pacing lead had penetrated was removed.

**Conclusions:**

The patient tolerated the procedure well and was discharged 1 week after the operation. This case adds to the current literature the justification of removal of temporary and non-functional pacing leads.

## Background

Perforation of a ventricular pacing lead through the heart is uncommon, but can result in a potentially lethal event such as cardiac tamponade. Twelve years ago, Singhal et al. described a right ventricular lead perforation which had eroded through a rib [1]. Similar to this report, a small rib resection was done through a chest wall exploration. The right ventricle was repaired with a purse-string suture. This group recommended that percutaneous lead extractions such as this should be done in the operating room with a general anesthesia and transesophageal echo monitoring in case a cardiac injury needs to be directly repaired [1]. This case report is only the second to include rib perforation with pacing lead migration. There are other reports that describe pacing lead migration into the chest wall including breast tissue [2] and even as far superior as the jaw [3]. We describe the presentation of a patient that had a right ventricular pacer lead erode through the left fourth rib and describe the surgical treatment of this patient. Although perforation of pacing lead through the right ventricular wall is a considerable event, we focus this report on the treatment of pacer lead eroding through the patient’s fourth rib.

## Case presentation

**Fig. 1:**
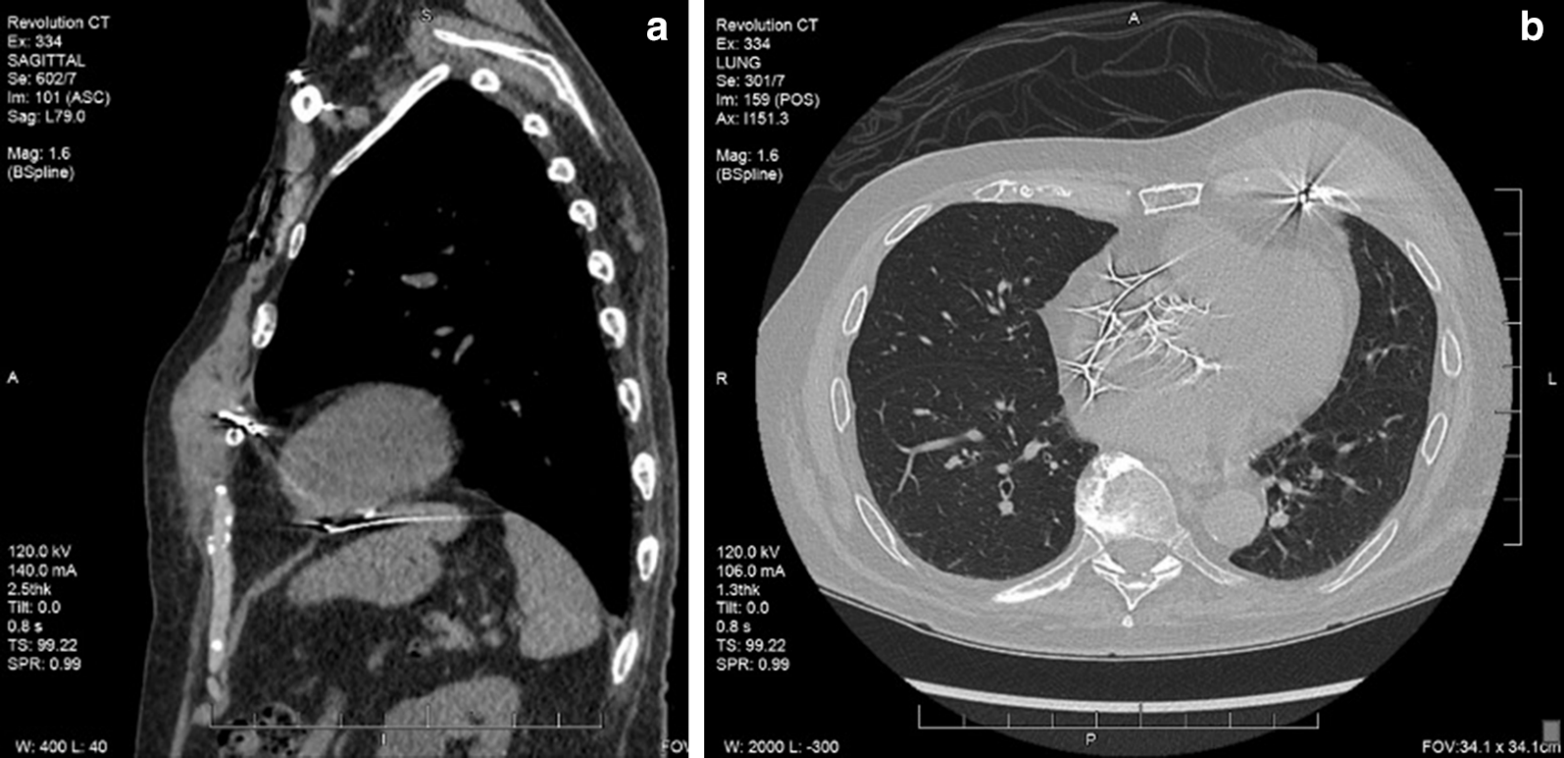
**a** Sagittal CT noting the ventricular lead rib perforation and anterior chest wall mass and **b** thoracic CT imaging the left anterior chest wall mass and erosion of ventricular pace lead into a rib

A 90-year-old male presented with a tender left chest wall mass and complete heart block. He complained of pain upon palpation of the mass (Fig. 1). Two years prior to presentation, the patient underwent a transfemoral aortic valve replacement and was subsequently dependent on a temporary pacemaker. Because the patient was dependent on a temporary pacemaker, a dual chamber pacemaker was placed 2 days after the aortic valve had been placed. A day after the dual chamber pacemaker had been placed, the ventricular screw-in pacing lead became non-functional and a new, temporary ventricular lead had to be placed. A chest radiograph revealed that the original, non-functional ventricular lead appeared to have perforated the myocardium. This non-functioning ventricular lead was left in place for fear that its removal would result in bleeding and cardiac tamponade. A CT scan showed that the original, non-functional ventricular lead which had perforated the right ventricle and was left in place, burrowed through the pericardial space, and produced a hematoma in the subcutaneous tissue of the left chest wall over the last 2 years (Fig. 1).

**Fig. 2:**
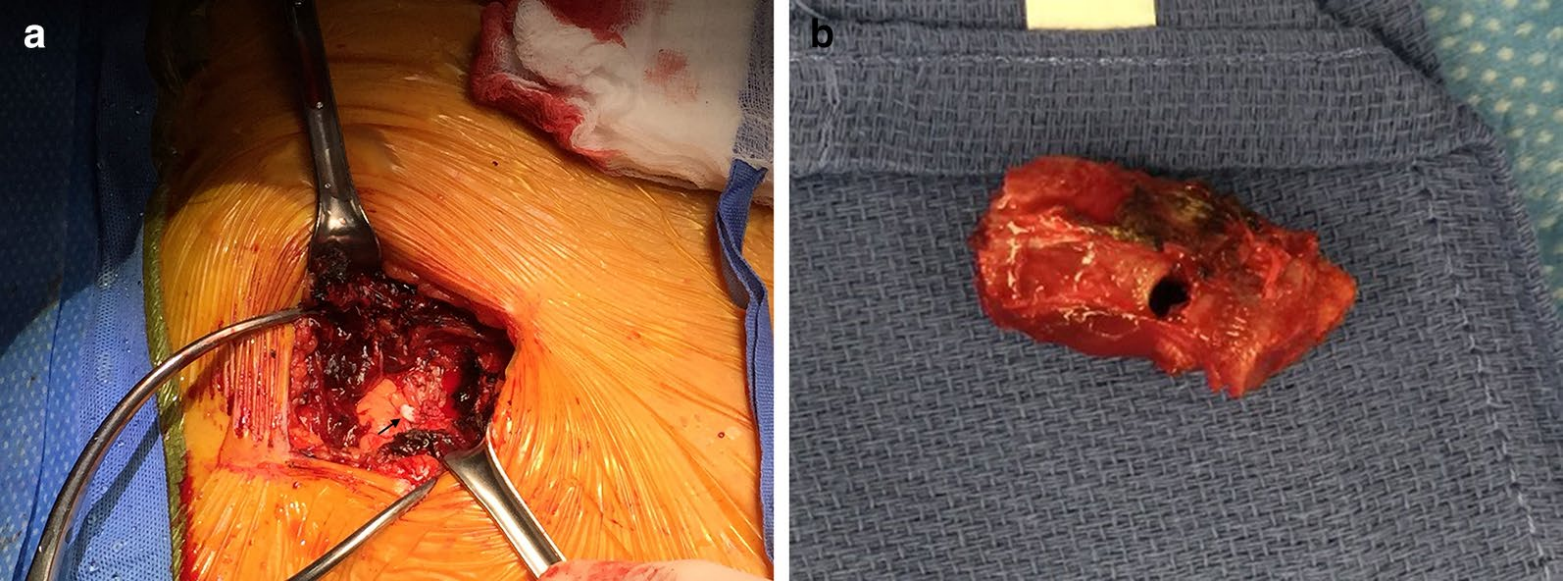
**a** Open thoracotomy view of the non-functional pace lead emerging through the fourth rib indicated with arrow and **b** removed portion of rib demonstrating erosion from pacing lead

The patient was prepped and draped for a possible median sternotomy and the heart–lung machine was placed on standby. A 10-cm oblique incision was made centered over the chest wall mass. The pectoralis muscle was divided, and the hematoma was evacuated (Fig. 2a). The tip of the original, non-functional lead that was left in place was found to have eroded through the fourth rib (Fig. 2a arrow). A segment of the fourth rib was removed (Fig. 2b). After the rib was removed, the non-functional pacing lead could be seen protruding through the wall of the exposed right ventricle. The area of the heart around the perforation was dissected free and two 3-0 pledgetted sutures were used to close the defect. The pacing lead was pulled and transected; about 6 cm of lead was excised. The remaining lead was capped and allowed to retract back into the right ventricle during the surgery. The defect in the ventricle was plugged with the previously placed prolene mattress sutures. The chest wound was closed in layers. The patient tolerated the procedure well and was discharged to home 1 week after the operation.

## Conclusions

Infection, inappropriate shocks, lead migration, vein thrombosis, insulation defects with inappropriate muscle stimulation, and acute cardiac perforation with pericardial effusion and tamponade are all possible complications of pacemaker and implantable cardioverter defibrillator (ICD) insertions. Delayed lead perforation is another known complication of pacemaker and ICD insertions and occurs in 0.1–0.8% of pacemaker and 0.6–5.2% if defibrillator insertions [4]. By definition, delayed perforations are those that occur more than 1 month after implantation [5]. These perforations can occur with both passive and active fixation leads. Loss of pacing, palpitations at the lead perforation site, and pericardial and pleural effusions can be associated with lead perforations, but many lead insertions may be asymptomatic. Relying on cardiologist or primary care providers to routinely follow patients that receive pacing leads is recommended to detect any asymptomatic changes.

The patient age, etiology, timing, and presenting symptoms of pace lead migration is unpredictable. In the Singhal case, the 50-year-old patient experienced a motor vehicle accident 7 years prior [1]. In this case report of a 90-year-old, and in the case presented by Ghazali et al. of a 13-year-old, the patients did not report any history of a traumatic event and presented 2 years after the pacing lead was placed [2]. Haq et al. described a case of late pacemaker lead perforation which manifested with new palpitations 2 weeks prior to the surgical repair in an 86-year-old patient [6]. The patient was well for 16 months after the initial pacemaker implantation [6]. We propose that heart mechanics are responsible for lead perforations. As the heart beats over time the tension against the endocardium may cause this type of lead to erode through to the myocardium or further. Since the patient in this report had a large subcutaneous hematoma, the decision was made to approach the injury through a median sternotomy. After a successful cardiac repair at the perforation site, a new transvenous lead was placed [6]. In an article from Montefiore Medical Center, an asymptomatic lead perforation is described [7]. Treatment options included lead withdrawal with cardiac surgery standby or lead withdrawal in the operating room. Because of the late presentation, they were concerned the lead had an endothelialized tract which would not self-seal so they chose the second treatment option [7].

Removal of a pacer lead that has eroded through the heart and into the chest wall can be done with an anterior thoracotomy so long as there is no evidence of a pericardial effusion or fluid in the chest. The lead exit site from the heart can be controlled with pledgetted sutures which can be secured after the lead is removed from the muscle. Heart–lung machine standby and prepping for open heart surgery is reasonable in case the ventricular perforation cannot be controlled with local means. If the lead perforation is associated with a large pericardial effusion, median sternotomy with pump standby is a reasonable approach for the needed cardiac repair of the perforation site. Since lead perforations can occur several months after implantation and be asymptomatic, it is reasonable to follow these leads on a periodic basis.

## Data Availability

Not applicable.

## Abbreviation

ICD: Implantable cardioverter defibrillator
